# Supplementary material for: Mapping the evolution of fertility support policies in China: A content and instrumental analysis
Source: PLoS One. 2025 Oct 9;20(10):e0332137. doi: 10.1371/journal.pone.0332137 (PMC12510515; doi:10.1371/journal.pone.0332137)
Supplement: S1 Appendix — (ZIP) [file pone.0332137.s001.zip › S1 Appendix. 226 original policy documents/137-中共中央、国务院关于新时代推进西部大开发形成新格局的指导意见(FBM-CLI-16-342306).doc]

中共中央、国务院关于新时代推进西部大开发形成新格局的指导意见

**发布部门：** [中国共产党中央委员会](https://www.pkulaw.com/law/chl?Aggs.IssueDepartment=a01) [国务院](https://www.pkulaw.com/law/chl?Aggs.IssueDepartment=5)

**发布日期：**2020.05.17

**实施日期：**2020.05.17

**时效性：** [现行有效](https://www.pkulaw.com/law/chl?Aggs.TimelinessDic=01)

**效力级别：** [党内法规制度](https://www.pkulaw.com/law/chl?Aggs.EffectivenessDic=XR12)

**法规类别：** [区域经济开发](https://www.pkulaw.com/law/chl?Aggs.Category=02308) [营商环境优化](https://dobusiness.pkulaw.com/)

中共中央、国务院关于新时代推进西部大开发形成新格局的指导意见

　　强化举措推进西部大开发形成新格局，是党中央、国务院从全局出发，顺应中国特色社会主义进入新时代、区域协调发展进入新阶段的新要求，统筹国内国际两个大局作出的重大决策部署。党的十八大以来，在以习近平同志为核心的党中央坚强领导下，西部地区经济社会发展取得重大历史性成就，为决胜全面建成小康社会奠定了比较坚实的基础，也扩展了国家发展的战略回旋空间。但同时，西部地区发展不平衡不充分问题依然突出，巩固脱贫攻坚任务依然艰巨，与东部地区发展差距依然较大，维护民族团结、社会稳定、国家安全任务依然繁重，仍然是全面建成小康社会、实现社会主义现代化的短板和薄弱环节。新时代继续做好西部大开发工作，对于增强防范化解各类风险能力，促进区域协调发展，决胜全面建成小康社会，开启全面建设社会主义现代化国家新征程，具有重要现实意义和深远历史意义。为加快形成西部大开发新格局，推动西部地区高质量发展，现提出如下意见。
　　一、总体要求
　　以习近平新时代中国特色社会主义思想为指导，全面贯彻党的十九大和十九届二中、三中全会精神，统筹推进“五位一体”总体布局，协调推进“四个全面”战略布局，落实总体国家安全观，坚持稳中求进工作总基调，坚持新发展理念，坚持推动高质量发展，坚持以供给侧结构性改革为主线，深化市场化改革、扩大高水平开放，坚定不移推动重大改革举措落实，防范化解推进改革中的重大风险挑战。强化举措抓重点、补短板、强弱项，形成大保护、大开放、高质量发展的新格局，推动经济发展质量变革、效率变革、动力变革，促进西部地区经济发展与人口、资源、环境相协调，实现更高质量、更有效率、更加公平、更可持续发展，确保到2020年西部地区生态环境、营商环境、开放环境、创新环境明显改善，与全国一道全面建成小康社会；到2035年，西部地区基本实现社会主义现代化，基本公共服务、基础设施通达程度、人民生活水平与东部地区大体相当，努力实现不同类型地区互补发展、东西双向开放协同并进、民族边疆地区繁荣安全稳固、人与自然和谐共生。

　　二、贯彻新发展理念，推动高质量发展
　　（一）打好三大攻坚战。把打好三大攻坚战特别是精准脱贫攻坚战作为决胜全面建成小康社会的关键任务，集中力量攻坚克难。重点解决实现“两不愁三保障”面临的突出问题，加大深度贫困地区和特殊贫困群体脱贫攻坚力度，减少和防止贫困人口返贫，确保到2020年现行标准下西部地区农村贫困人口全部实现脱贫，贫困县全部摘帽。在全面完成脱贫任务基础上压茬推进乡村振兴战略，巩固脱贫攻坚成果。结合西部地区发展实际，打好污染防治标志性重大战役，实施环境保护重大工程，构建生态环境分区管控体系。精准研判可能出现的主要风险点，结合西部地区实际，进一步完善体制机制，拿出改革创新举措。坚持底线思维，强化源头管控，有效稳住杠杆率。
　　（二）不断提升创新发展能力。以创新能力建设为核心，加强创新开放合作，打造区域创新高地。完善国家重大科研基础设施布局，支持西部地区在特色优势领域优先布局建设国家级创新平台和大科学装置。加快在西部具备条件的地区创建国家自主创新示范区、科技成果转移转化示范区等创新载体。进一步深化东西部科技创新合作，打造协同创新共同体。在西部地区布局建设一批应用型本科高校、高职学校，支持“双一流”高校对西部地区开展对口支援。深入推进大众创业万众创新，促进西部地区创新创业高质量发展，打造“双创”升级版。健全以需求为导向、以企业为主体的产学研一体化创新体制，鼓励各类企业在西部地区设立科技创新公司。支持国家科技成果转化引导基金在西部地区设立创业投资子基金。加强知识产权保护、应用和服务体系建设，支持开展知识产权国际交流合作。
　　（三）推动形成现代化产业体系。充分发挥西部地区比较优势，推动具备条件的产业集群化发展，在培育新动能和传统动能改造升级上迈出更大步伐，促进信息技术在传统产业广泛应用并与之深度融合，构建富有竞争力的现代化产业体系。推动农村一二三产业深度融合，促进农牧业全产业链、价值链转型升级。加快推进高标准农田、现代化生态牧场、粮食生产功能区和棉油糖等重要农产品生产保护区建设，支持发展生态集约高效、用地规范的设施农业。加快高端、特色农机装备生产研发和推广应用。推动发展现代制造业和战略性新兴产业。积极发展大数据、人工智能和“智能＋”产业，大力发展工业互联网。推动“互联网＋教育”、“互联网＋医疗”、“互联网＋旅游”等新业态发展，推进网络提速降费，加快发展跨境电子商务。支持西部地区发挥生态、民族民俗、边境风光等优势，深化旅游资源开放、信息共享、行业监管、公共服务、旅游安全、标准化服务等方面国际合作，提升旅游服务水平。依托风景名胜区、边境旅游试验区等，大力发展旅游休闲、健康养生等服务业，打造区域重要支柱产业。加快发展现代服务业特别是专业服务业，加强现代物流服务体系建设。
　　（四）优化能源供需结构。优化煤炭生产与消费结构，推动煤炭清洁生产与智能高效开采，积极推进煤炭分级分质梯级利用，稳步开展煤制油、煤制气、煤制烯烃等升级示范。建设一批石油天然气生产基地。加快煤层气等勘探开发利用。加强可再生能源开发利用，开展黄河梯级电站大型储能项目研究，培育一批清洁能源基地。加快风电、光伏发电就地消纳。继续加大西电东送等跨省区重点输电通道建设，提升清洁电力输送能力。加强电网调峰能力建设，有效解决弃风弃光弃水问题。积极推进配电网改造行动和农网改造升级，提高偏远地区供电能力。加快北煤南运通道和大型煤炭储备基地建设，继续加强油气支线、终端管网建设。构建多层次天然气储备体系，在符合条件的地区加快建立地下储气库。支持符合环保、能效等标准要求的高载能行业向西部清洁能源优势地区集中。
　　（五）大力促进城乡融合发展。深入实施乡村振兴战略，做好新时代“三农”工作。培养新型农民，优化西部地区农业从业者结构。以建设美丽宜居村庄为目标，加强农村人居环境和综合服务设施建设。在加强保护基础上盘活农村历史文化资源，形成具有地域和民族特色的乡村文化产业和品牌。因地制宜优化城镇化布局与形态，提升并发挥国家和区域中心城市功能作用，推动城市群高质量发展和大中小城市网络化建设，培育发展一批特色小城镇。加大对西部地区资源枯竭等特殊类型地区振兴发展的支持力度。有序推进农业转移人口市民化。推动基本公共服务常住人口全覆盖，保障符合条件的未落户农民工在流入地平等享受城镇基本公共服务。总结城乡“资源变资产、资金变股金、农（市）民变股东”等改革经验，探索“联股联业、联股联责、联股联心”新机制。统筹城乡市政公用设施建设，促进城镇公共基础设施向周边农村地区延伸。
　　（六）强化基础设施规划建设。提高基础设施通达度、通畅性和均等化水平，推动绿色集约发展。加强横贯东西、纵贯南北的运输通道建设，拓展区域开发轴线。强化资源能源开发地干线通道规划建设。加快川藏铁路、沿江高铁、渝昆高铁、西（宁）成（都）铁路等重大工程规划建设。注重高速铁路和普通铁路协同发展，继续开好多站点、低票价的“慢火车”。打通断头路、瓶颈路，加强出海、扶贫通道和旅游交通基础设施建设。加强综合客运枢纽、货运枢纽（物流园区）建设。完善国家物流枢纽布局，提高物流运行效率。加强航空口岸和枢纽建设，扩大枢纽机场航权，积极发展通用航空。进一步提高农村、边远地区信息网络覆盖水平。合理规划建设一批重点水源工程、江河湖泊骨干治理工程和大型灌区工程，加强大中型灌区续建配套与现代化改造、中小河流治理和病险水库除险加固、抗旱水源工程建设和山洪灾害防治。推进城乡供水一体化和人口分散区域重点小型标准化供水设施建设，加强饮用水水源地规范化建设。
　　（七）切实维护国家安全和社会稳定。统筹发展与安全两件大事，更好发挥西部地区国家安全屏障作用。巩固和发展平等团结互助和谐的社会主义民族关系，促进各民族共同团结奋斗和共同繁荣发展。深入推进立体化社会治安防控，构建坚实可靠的社会安全体系。

　　三、以共建“一带一路”为引领，加大西部开放力度
　　（八）积极参与和融入“一带一路”建设。支持新疆加快丝绸之路经济带核心区建设，形成西向交通枢纽和商贸物流、文化科教、医疗服务中心。支持重庆、四川、陕西发挥综合优势，打造内陆开放高地和开发开放枢纽。支持甘肃、陕西充分发掘历史文化优势，发挥丝绸之路经济带重要通道、节点作用。支持贵州、青海深化国内外生态合作，推动绿色丝绸之路建设。支持内蒙古深度参与中蒙俄经济走廊建设。提升云南与澜沧江－湄公河区域开放合作水平。
　　（九）强化开放大通道建设。积极实施中新（重庆）战略性互联互通示范项目。完善北部湾港口建设，打造具有国际竞争力的港口群，加快培育现代海洋产业，积极发展向海经济。积极发展多式联运，加快铁路、公路与港口、园区连接线建设。强化沿江铁路通道运输能力和港口集疏运体系建设。依托长江黄金水道，构建陆海联运、空铁联运、中欧班列等有机结合的联运服务模式和物流大通道。支持在西部地区建设无水港。优化中欧班列组织运营模式，加强中欧班列枢纽节点建设。进一步完善口岸、跨境运输和信息通道等开放基础设施，加快建设开放物流网络和跨境邮递体系。加快中国－东盟信息港建设。
　　（十）构建内陆多层次开放平台。鼓励重庆、成都、西安等加快建设国际门户枢纽城市，提高昆明、南宁、乌鲁木齐、兰州、呼和浩特等省会（首府）城市面向毗邻国家的次区域合作支撑能力。支持西部地区自由贸易试验区在投资贸易领域依法依规开展先行先试，探索建设适应高水平开放的行政管理体制。加快内陆开放型经济试验区建设，研究在内陆地区增设国家一类口岸。研究按程序设立成都国际铁路港经济开发区。有序推进国家级新区等功能平台建设。整合规范现有各级各类基地、园区，加快开发区转型升级。鼓励国家级开发区实行更加灵活的人事制度，引进发展优质医疗、教育、金融、物流等服务。办好各类国家级博览会，提升西部地区影响力。
　　（十一）加快沿边地区开放发展。完善沿边重点开发开放试验区、边境经济合作区、跨境经济合作区布局，支持在跨境金融、跨境旅游、通关执法合作、人员出入境管理等方面开展创新。扎实推进边境旅游试验区、跨境旅游合作区、农业对外开放合作试验区等建设。统筹利用外经贸发展专项资金支持沿边地区外经贸发展。完善边民互市贸易管理制度。深入推进兴边富民行动。
　　（十二）发展高水平开放型经济。推动西部地区对外开放由商品和要素流动型逐步向规则制度型转变。落实好外商投资准入前国民待遇加负面清单管理制度，有序开放制造业，逐步放宽服务业准入，提高采矿业开放水平。支持西部地区按程序申请设立海关特殊监管区域，支持区域内企业开展委内加工业务。加强农业开放合作。推动西部优势产业企业积极参与国际产能合作，在境外投资经营中履行必要的环境、社会和治理责任。支持建设一批优势明显的外贸转型升级基地。建立东中西部开放平台对接机制，共建项目孵化、人才培养、市场拓展等服务平台，在西部地区打造若干产业转移示范区。对向西部地区梯度转移企业，按原所在地区已取得的海关信用等级实施监督。
　　（十三）拓展区际互动合作。积极对接京津冀协同发展、长江经济带发展、粤港澳大湾区建设等重大战略。支持青海、甘肃等加快建设长江上游生态屏障，探索协同推进生态优先、绿色发展新路径。依托陆桥综合运输通道，加强西北省份与江苏、山东、河南等东中部省份互惠合作。加快珠江－西江经济带和北部湾经济区建设，鼓励广西积极参与粤港澳大湾区建设和海南全面深化改革开放。推动东西部自由贸易试验区交流合作，加强协同开放。支持跨区域共建产业园区，鼓励探索“飞地经济”等模式。加强西北地区与西南地区合作互动，促进成渝、关中平原城市群协同发展，打造引领西部地区开放开发的核心引擎。推动北部湾、兰州－西宁、呼包鄂榆、宁夏沿黄、黔中、滇中、天山北坡等城市群互动发展。支持南疆地区开放发展。支持陕甘宁、川陕、左右江等革命老区和川渝、川滇黔、渝黔等跨省（自治区、直辖市）毗邻地区建立健全协同开放发展机制。加快推进重点区域一体化进程。

　　四、加大美丽西部建设力度，筑牢国家生态安全屏障
　　（十四）深入实施重点生态工程。坚定贯彻绿水青山就是金山银山理念，坚持在开发中保护、在保护中开发，按照全国主体功能区建设要求，保障好长江、黄河上游生态安全，保护好冰川、湿地等生态资源。进一步加大水土保持、天然林保护、退耕还林还草、退牧还草、重点防护林体系建设等重点生态工程实施力度，开展国土绿化行动，稳步推进自然保护地体系建设和湿地保护修复，展现大美西部新面貌。加快推进国家公园体系建设。
　　（十五）稳步开展重点区域综合治理。大力推进青海三江源生态保护和建设、祁连山生态保护与综合治理、岩溶地区石漠化综合治理、京津风沙源治理等。以汾渭平原、成渝地区、乌鲁木齐及周边地区为重点，加强区域大气污染联防联控，提高重污染天气应对能力。开展西部地区土壤污染状况详查，积极推进受污染耕地分类管理和安全利用，有序推进治理与修复。
　　（十六）加快推进西部地区绿色发展。落实市场导向的绿色技术创新体系建设任务，推动西部地区绿色产业加快发展。实施国家节水行动以及能源消耗总量和强度双控制度，全面推动重点领域节能减排。大力发展循环经济，推进资源循环利用基地建设和园区循环化改造，鼓励探索低碳转型路径。全面推进河长制、湖长制，推进绿色小水电改造。加快西南地区城镇污水管网建设和改造，加强入河排污口管理，强化西北地区城中村、老旧城区和城乡结合部污水截流、收集、纳管工作。加强跨境生态环境保护合作。

　　五、深化重点领域改革，坚定不移推动重大改革举措落实
　　（十七）深化要素市场化配置改革。探索集体荒漠土地市场化路径，设定土地用途，鼓励个人申领使用权。深入推进主业为充分竞争行业的商业类地方国有企业混合所有制改革。深化资源性产品等要素价格形成机制改革，建立健全定价成本信息公开制度。有序放开竞争性环节电价，深化输配电价改革。推进增量配电业务改革试点，开展电力现货交易试点。实施丰水期居民生活电能替代等电价政策，促进西部地区清洁能源消纳。建立健全天然气弹性价格机制和上下游价格传导机制。建立健全市场化、多元化生态保护补偿机制，进一步完善生态保护补偿市场体系。构建统一的自然资源资产交易平台，健全自然资源资产收益分配制度。提高西部地区直接融资比例，支持符合条件的企业在境内外发行上市融资、再融资，通过发行公司信用类债券、资产证券化产品融资。西部贫困地区企业首次公开发行上市、新三板挂牌、发行债券、并购重组等适用绿色通道政策。
　　（十八）积极推进科技体制改革。开展探索赋予科研人员职务科技成果所有权或长期使用权试点工作。支持扩大科研经费使用自主权，提高智力密集型项目间接经费比例并向创新绩效突出的团队和个人倾斜。加快科技人员薪酬制度改革，扩大高校和科研院所工资分配自主权，健全绩效工资分配机制。
　　（十九）持续推进信用体系建设。建立健全地方信用法规体系。加强政务诚信建设，建立健全政府失信责任追究制度。完善省市县信用信息共享平台。加快征信市场建设，培育有良好信誉的信用服务机构，鼓励研发适合西部地区的征信产品。
　　（二十）努力营造良好营商环境。深化“放管服”改革，加快建设服务型政府。落实全国统一的[市场准入负面清单](https://www.pkulaw.com/chl/fb62cc1e71e4ee0bbdfb.html)制度，推动“非禁即入”普遍落实。推行政务服务“最多跑一次”和企业投资项目承诺制改革，大幅压缩工程建设项目审批时间。落实减税降费各项政策措施，着力降低物流、用能等费用。实施“双随机、一公开”监管，对新技术、新业态、新模式实行审慎包容监管，提高监管效能，防止任意检查、执法扰民。强化竞争政策的基础性地位，进一步落实公平竞争审查制度，加快清理废除妨碍统一市场和公平竞争的各种规定和做法，持续深入开展不正当竞争行为治理，形成优化营商环境长效机制。

　　六、坚持以人民为中心，把增强人民群众获得感、幸福感、安全感放到突出位置
　　（二十一）着力强化公共就业创业服务。完善城乡劳动者终身职业技能培训政策和组织实施体系。强化就业和国家通用语言培训。加大对高校毕业生在西部地区就业的扶持力度。积极引导农村劳动力转移就业和农民工返乡创业就业。妥善做好化解过剩产能中的职工分流安置工作。加大力度支持灵活就业和新就业形态。
　　（二十二）支持教育高质量发展。加强普惠性幼儿园建设，大力培养培训贫困地区幼儿园教师。加快改善贫困地区义务教育薄弱学校基本办学条件，全面加强乡村小规模学校、乡镇寄宿制学校建设。在县域义务教育学校学位供需矛盾突出地区有序增加义务教育供给，有效解决“大班额”问题，做好控辍保学工作。发展现代职业教育，推进职业教育东西协作，促进产教融合、校企合作。逐步普及高中阶段教育。加强学校语言文字工作，确保国家通用语言文字作为教育教学基本用语用字。支持探索利用人工智能、互联网开展远程教育，促进优质教学资源共享。支持西部地区高校“双一流”建设，着力加强适应西部地区发展需求的学科建设。持续推动东西部地区教育对口支援，继续实施东部地区高校对口支援西部地区高校计划、国家支援中西部地区招生协作计划，实施东部地区职业院校对口西部职业院校计划。促进西部高校国际人才交流，相关人才引进平台建设向西部地区倾斜。鼓励支持部委属高校和地方高校“订单式”培养西部地区专业化人才。
　　（二十三）提升医疗服务能力和水平。重点加强西部地区县级（含兵团团场）医院综合能力建设，持续改善农村医疗卫生条件，加快基层医疗卫生机构标准化建设。改善医疗基础设施和装备条件，提高医护人员专业技术水平。支持在西部地区建立若干区域医疗中心。探索利用人工智能、互联网等开展远程医疗，支持宁夏建设“互联网＋医疗健康”示范区。充分发挥中医药在医疗卫生服务中的作用。加快补齐3岁以下婴幼儿照护服务短板。支持西部地区医疗机构与东中部地区医疗机构间开展双向交流。
　　（二十四）完善多层次广覆盖的社会保障体系。加快推进养老保险省级统筹，推进落实城乡居民基本养老保险待遇确定和基础养老金正常调整机制。合理确定基本医疗保险保障水平，完善医疗保险关系转移接续措施。完善失业保险制度，逐步提高失业保障水平。科学制定低保标准，逐步拓展低保覆盖范围。建设统一的社会保险公共服务平台，推广以社会保障卡为载体的“一卡通”服务管理模式。
　　（二十五）健全养老服务体系。加快构建以居家为基础、社区为依托、机构为补充、医养相结合的养老服务体系。稳步推进公办养老机构改革和建设，全面放开养老服务市场，积极引导社会资本进入养老服务业，扩大西部地区养老服务有效供给，探索建立长期照护保障体系。加大对养老服务设施建设支持力度，加强农村特困人员供养服务机构建设管理，稳步提高托底保障能力和服务质量。实施养老服务专业人才培养等工程。
　　（二十六）强化公共文化体育服务。完善公共文化服务设施网络，强化数字技术运用，推动文化惠民工程整合创新、提挡升级。推进县级融媒体中心建设，推动广播电视户户通，建立健全应急广播平台及传输覆盖网络。鼓励发展含少数民族传统体育在内的群众体育。加强公共体育场馆建设，推进相关场馆免费或低收费开放。
　　（二十七）改善住房保障条件。完善分类分级补助标准，加大对农村危房改造补助资金倾斜支持力度。鼓励通过闲置农房置换或长期租赁等方式，解决农村特困群体基本住房安全问题。落实易地扶贫搬迁政策，完善安置区配套基础设施和公共服务设施。积极改善城镇中等偏下及以下收入住房困难家庭、新就业无房职工和城镇稳定就业的无房外来务工人员居住条件。
　　（二十八）增强防灾减灾与应急管理能力。推进西部地区城乡基层防灾减灾救灾能力建设，完善事故灾害综合风险评估技术标准体系，推进事故灾害综合风险评估和隐患排查治理。结合西部地区实际，推进实施灾害风险防控、监测预警、应急抢险救援、信息服务保障、救灾物资储备以及防灾减灾救灾科技支撑、宣传教育等能力建设工程。实施地震易发区房屋设施加固工程。推进西部地区灾害应急救援联动指挥平台建设，建立应急救援资源共享及联合处置机制。打造符合西部地区需求的防灾减灾救灾科技创新团队、实验基地和实验平台。加快提高骨干救援队伍专业化技术装备水平。

　　七、加强政策支持和组织保障
　　（二十九）分类考核。参照高质量发展综合评价指标和分领域评价指标，根据西部地区不同地域特点，设置各有侧重、各具特色的考核内容和指标，实施差异化考核。深入研究制定分类考核的具体措施。
　　（三十）财税支持。稳妥有序推进中央和地方收入划分改革。中央财政在一般性转移支付和各领域专项转移支付分配中，继续通过加大资金分配系数、提高补助标准或降低地方财政投入比例等方式，对西部地区实行差别化补助，加大倾斜支持力度。考虑重点生态功能区占西部地区比例较大的实际，继续加大中央财政对重点生态功能区转移支付力度，完善资金测算分配办法。考虑西部地区普遍财力较为薄弱的实际，加大地方政府债券对基础设施建设的支持力度，将中央财政一般性转移支付收入纳入地方政府财政承受能力计算范畴。指导推动省以下财政事权和支出责任划分，调动市县积极性。对设在西部地区的鼓励类产业企业所得税优惠等政策到期后继续执行。赋予西部地区具备条件且有需求的海关特殊监管区域内企业增值税一般纳税人资格。对西部地区鼓励类产业项目在投资总额内进口的自用设备，在政策规定范围内免征关税。
　　（三十一）金融支持。支持商业金融、合作金融等更好为西部地区发展服务。引导金融机构加大对西部地区小微企业融资支持力度。落实无还本续贷、尽职免责等监管政策，在风险总体可控前提下加大对西部地区符合条件的小微企业续贷支持力度。引导和鼓励银行业金融机构合理调配信贷资源，加大对西部贫困地区扶贫产业支持力度。支持轻资产实体经济企业或项目以适当方式融资。增加绿色金融供给，推动西部地区经济绿色转型升级。依法合规探索建立西部地区基础设施领域融资风险分担机制。
　　（三十二）产业政策。实行负面清单与鼓励类产业目录相结合的产业政策，提高政策精准性和精细度。在执行全国统一的[市场准入负面清单](https://www.pkulaw.com/chl/fb62cc1e71e4ee0bbdfb.html)基础上，对[西部地区鼓励类产业目录](https://www.pkulaw.com/chl/30962eaeef746232bdfb.html)进行动态调整，与分类考核政策相适应。适时修订[中西部地区外商投资优势产业目录](https://www.pkulaw.com/chl/c2eb5f655fba5647bdfb.html)并进行动态调整。继续完善产业转移引导政策，适时更新[产业转移指导目录](https://www.pkulaw.com/chl/62fe97da1ec44a4fbdfb.html)。加大中央财政对西部地区自然资源调查评价的支持力度，自然资源调查计划优先安排西部地区项目。凡有条件在西部地区就地加工转化的能源、资源开发利用项目，支持在当地优先布局建设并优先审批核准。鼓励新设在西部地区的中央企业及其分支机构在当地注册。适当降低社会保险费率，确保总体上不增加企业负担。
　　（三十三）用地政策。继续实施差别化用地政策，新增建设用地指标进一步向西部地区倾斜，合理增加荒山、沙地、戈壁等未利用土地开发建设指标。加强对基础设施领域补短板项目的用地保障。支持西部地区开放平台建设，对国家级新区、开发区利用外资项目以及重点开发开放试验区、边境经济合作区、跨境经济合作区产业发展所需建设用地，在计划指标安排上予以倾斜支持。推进耕地指标和城乡建设用地指标在国家统筹管理下实现跨省域调剂。
　　（三十四）人才政策。努力造就忠诚干净担当的西部地区高素质干部队伍，注重选拔符合西部地区需要的专业化人才，建立健全有利于吸引、激励和留住人才的体制机制。落实完善工资待遇倾斜政策，结合事业单位改革，鼓励引导机关事业单位人员特别是基层公务员、教师、医护人员、科技人员等扎根西部。鼓励符合条件的企业实施股权激励、分红等中长期激励。允许国有企事业单位专业技术和管理人才按有关规定在西部地区兼职并取得合法报酬。允许退休公职人员按有关规定在西部地区创业。
　　（三十五）帮扶政策。深入开展对口支援新疆、西藏和青海等省藏区以及对口帮扶贵州等工作。继续实施中央和国家机关及企事业单位等定点帮扶。支持军队发挥优势，积极参与西部大开发。推动统一战线继续支持毕节试验区改革发展。鼓励东中部城市帮助边境城市对口培训亟需的管理和技术人才。鼓励企业结对帮扶贫困县（村）。进一步推动从中央和国家机关、东部地区选派优秀干部到西部地区挂职任职，注重提拔使用在西部地区作出突出贡献的优秀干部。继续做好公务员对口培训工作。
　　（三十六）组织保障。加强党对西部大开发工作的领导，强化各级党组织在推进西部大开发形成新格局进程中的领导作用。强化基层党组织建设，健全以党组织为领导的组织体系，着力提升基层党组织的组织力，引导广大党员干部在西部大开发中发挥先锋模范作用。激励干部担当作为，鼓励创造性贯彻落实。国务院西部地区开发领导小组要加强统筹指导，各成员单位和有关部门要各司其职、压实责任，密切配合、通力协作，制定配套政策措施并推进落实。国家发展改革委要切实承担国务院西部地区开发领导小组办公室职责，适时对政策实施情况进行评估，发挥好督查促落实作用。西部地区各级党委和政府要切实承担主体责任，主动作为、真抓实干，结合本地区实际出台贯彻落实本意见的具体举措，团结带领广大干部群众认真抓好各项任务落实。要切实解决困扰基层的形式主义问题，让西部地区基层干部腾出更多精力干实事。东中部地区及社会各界要继续支持和参与西部大开发。
　　各地区各部门要在以习近平同志为核心的党中央坚强领导下，增强“四个意识”，坚定“四个自信”，做到“两个维护”，认真落实党中央、国务院决策部署，解放思想、锐意进取、深化改革、破解矛盾，加快建立更加有效的区域协调发展新机制，以更大力度、更强举措推进西部大开发形成新格局。

　　（新华社北京2020年5月17日电）

© 北大法宝：（[www.pkulaw.com](https://www.pkulaw.com/)) 专业提供法律信息、法学知识和法律软件领域各类解决方案。北大法宝为您提供丰富的参考资料，正式引用法规条文时请与标准文本核对。
欢迎查看所有[产品和服务](https://www.pkulaw.com/helps/2.html)。法宝快讯：[如何快速找到您需要的检索结果？](https://www.pkulaw.com/helps/70.html)[法宝](https://www.pkulaw.com/helps/69.html)V6有何新特色？

扫描二维码阅读原文

**原文链接：**<https://www.pkulaw.com/chl/cb1bc501df35addbbdfb.html>
